# Use of the bacteriophage-derived endolysin CHAPK-SH3blys as a potent novel treatment for biofilm-associated *Staphylococcus aureus* wound infections

**DOI:** 10.1128/spectrum.00716-25

**Published:** 2025-08-05

**Authors:** Hawraa Shahrour, Daniela Alves Ferreira, Deirdre Fitzgerald-Hughes, James P. O'Gara, Aidan Coffey, Eoghan O'Neill

**Affiliations:** 1 Department of Clinical Microbiology, RCSI Education & Research Centre, Beaumont Hospital, RCSI University of Medicine and Health Sciences 8863, Dublin, Ireland; 2 Department of Microbiology, School of Biological and Chemical Sciences, University of Galway 219560 , Galway, Ireland; 3 Department of Biological Sciences, Munster Technological University 415374, Cork, Ireland; 4 Department of Microbiology, Connolly Hospital 58867, Blanchardstown, Dublin, Ireland; Meijo University, Nagoya, Japan

**Keywords:** CHAPK-SH3blys, biofilm, endolysin, wound infections, *S. aureus*

## Abstract

The pathogenesis of *Staphylococcus aureus* acute and chronic wound infections is associated with biofilm formation. This study investigated the antimicrobial and antibiofilm potential of CHAPK-SH3blys, a bacteriophage-derived endolysin, against *S. aureus* strains implicated in chronic wound infections. CHAPK-SH3blys exhibited potent antibacterial activity with a minimum inhibitory concentration (MIC) of 3.9 µg/mL. Biofilm assays conducted under static and dynamic wound-like conditions showed a concentration-dependent reduction in biofilm metabolic activity, with up to a 4-log reduction in viable cells at concentrations 50 times the MIC. Further investigations revealed that CHAPK-SH3blys showed biocompatibility with human cell lines and did not induce an inflammatory response. Notably, prolonged exposure to sub-MIC levels did not lead to resistance development in *S. aureus*. These findings highlight CHAPK-SH3blys as a promising therapeutic agent for the treatment of biofilm-associated *S. aureus* wound infections.

**IMPORTANCE:**

Successful treatment of wound infections, which is most commonly caused by the bacteria *Staphylococcus aureus*, is compromised by bacterial colonization resulting in slime layer or biofilm formation in the wound bed. Associated antimicrobial resistance (AMR) and limited treatment options can also impair wound healing and further highlight the need for the development and use of novel antimicrobial therapies. This study explores the antibiofilm and antimicrobial effectiveness of a bacteriophage-derived endolysin named CHAPK-SH3blys against *S. aureus* strains implicated in chronic wound infections. Bacteriophage-derived endolysin represents novel antimicrobial agents, and in this study, we demonstrate the effectiveness of this agent in treating wound and biofilm- related infections under wound-like conditions in the laboratory without affecting the immune response or inducing antimicrobial resistance. Due to the urgent public health need to develop alternative antimicrobial agents to combat biofilm-associated infections, our study highlights CHAPK-SH3blys as a promising and exciting candidate for further therapeutic development.

## INTRODUCTION

*Staphylococcus aureus* is a Gram-positive bacterium commonly found colonizing the skin and mucous membranes of about 30% of the human population without causing disease (1). However, it is a frequent cause of various infections, ranging from minor skin and soft tissue infections (such as impetigo, cellulitis, abscesses, and wound infections) to severe systemic conditions such as pneumonia, osteomyelitis, septic arthritis, endocarditis, bacteremia, and sepsis (2–6). *S. aureus* is also recognized for its ability to develop resistance to different antibiotic classes (7).

Chronic wounds, such as diabetic foot ulcers and pressure sores, are also prone to infection by *S. aureus* due to their slow healing process, which allows bacterial colonization and infection (8, 9). The ability of *S. aureus* to form biofilms within the wound environment increases its resistance to the immune system clearance and conventional antibiotics. Additionally, *S. aureus* produces toxins and enzymes, such as proteases, lipases, and alpha-toxins that damage tissues and hinder healing, leading to complications like osteomyelitis (10). As a result, treating chronic wounds infected with *S. aureus* typically requires extended and specialized therapeutic approaches, which increase healthcare costs and make patient recovery more challenging (11, 12).

Advanced local therapeutic strategies for managing chronic wounds include the application of topical antimicrobial agents, such as silver-based dressings, honey, and iodine, which are employed to reduce bacterial load. In cases of infection, systemic antibiotics may also be necessary. Emerging treatments are increasing focus on biofilm-targeting approaches, utilizing biofilm-disrupting agents and enzymes to degrade the extracellular matrix and enhance antibiotic penetration. Given the global rise in antibiotic resistance, research groups have continued to investigate novel antimicrobials; such compounds include endolysins which are investigated in this study. In recent years, bacteriophage endolysins (phage lysins) have been the focus of research into combating antibiotic resistance in Gram-positive pathogens (13, 14). These peptidoglycan hydrolases possess a number of advantages over conventional antibiotics including rapid lytic activity against bacterial cells, low probability of developing bacterial resistance, and significantly lower chance of disrupting commensal microflora due to enzyme specificity. Endolysins also have a number of advantages over using whole bacteriophages as antimicrobial agents. In the case of whole bacteriophages, resistance arising from adsorption inhibition, restriction modification, and abortive infection has been reported in many genera (15); in contrast, for endolysins, there has been no report of bacteria developing resistance even after the extensive growth of the bacterium in the presence of sub lethal levels of enzyme (16). Endolysins have also shown considerable promise in combating biofilm-associated infections, with numerous studies demonstrating significant reductions in *S. aureus* biofilms *in vitro* (17, 18). Previous data were reported on the use of the CHAPk staphylococcal phage endolysin to enhance antibacterial activity against stationary-phase cells (19). Here, we further investigated the antimicrobial potential and safety profile of the CHAPK-SH3blys endolysin as a potential novel treatment agent for *S. aureus* biofilm-related wound infections.

## MATERIALS AND METHODS

### Antimicrobials compounds

Endolysin CHAPK-SH3blys (truncated derivate of the phage K endolysin lysK) was obtained from Genoscript. The sequence coding CHAPK-SH3blys was obtained from sequence accession number AY176327.1. CHAPK-SH3blys was suspended in 25 mM Tris-HCl pH 7 buffer. Fusidic acid sodium salt and gentamicin were obtained from Merck (Ireland), and mupirocin was obtained from SLS (Ireland).

### Bacterial strains and growth conditions

Several bacterial strains were used, including methicillin-resistant *Staphylococcus aureus* (MRSA) strains USA300 LAC and BH1CC (20), methicillin-susceptible *S. aureus* (MSSA) strains SH1000 (21) and BH48 (20), as well as a gram-negative strain, *Pseudomonas aeruginosa* PAO1 (ATCC 156920). Bacteria were grown aerobically at 37°C with agitation at 200 rpm in Brain Heart Infusion (BHI, Oxoid, Ireland). For static antibiofilm assays simulating *in vivo* conditions, Bolton Broth (Fannin, Ireland) was enriched with 50% bovine heparin plasma (TebuBio, UK) and 5% laked horse blood (Merck, Ireland). For dynamic biofilm formation, Simulated Wound Fluid (SWF) was prepared following previous protocols with slight adjustments, using 3% (vol/vol) fetal bovine serum in maximum recovery diluent (Merck, Ireland) (22).

### Antimicrobial susceptibility testing

Minimal inhibitory concentrations (MIC) were determined using the standard broth microdilution method according to the guidelines by Clinical and Laboratory Standards Institute (CLSI) (23). Briefly, stock solutions of CHAPK-SH3blys, fusidic acid, gentamicin, and mupirocin were twofold serial diluted in cation adjusted Mueller-Hinton broth (CAMHB) (Merck, Ireland) in a 96-well plate round bottom polystyrene plates (ThermoFisher, Ireland). Bacterial suspensions were prepared in sterile PBS (Merck, Ireland) from isolated colonies grown on overnight plate cultures, to the density of a 0.5 McFarland standard (approximately 10^8^ colony-forming units [CFU]/mL) using a Densichek Meter (bioMèrieux). Afterward, bacterial suspensions were inoculated in CAMHB with antimicrobial compounds to a final concentration of 10^5^ CFU/mL. Microplates were incubated statically for 24 h at 37°C. Inoculated medium without antimicrobials served as growth controls and bacteria-free medium served as sterility controls. The lowest concentration of a compound or antibiotic showing no visible growth was recorded as the MIC. Experiments were conducted in triplicate, and independent replicates were performed three times in different days.

### Biofilm treatment with CHAPK-SH3blys under static conditions

Biofilms of *S. aureus* were established under static conditions using flat 96-well polystyrene plates, following the method outlined by Christensen et al. (24). In brief, bacterial suspensions were prepared from overnight cultures in Bolton Broth media supplemented with 50% bovine heparin plasma and 5% laked horse blood. Subsequently, 100 µL of each suspension was inoculated into the wells of the microtiter plates, followed by static incubation at 37°C for 1, 3, and 5 days. Daily replacement of the media was carried out for mature biofilms (at 3 and 5 days). Following the initial incubation and washing steps, treatment with CHAPK-SH3blys at concentrations ranging from 0.25 to 1 µg/mL was added to each test well, and the plates were further incubated at 37°C for 24 h. Post-treatment, the biofilms underwent two washes with PBS, followed by investigation of metabolic activity (Resazarin conversion) and viability (colony counting) as described below. Fresh media devoid of bacteria served as a negative control and remained in the wells during the incubation period. The experiments were conducted in triplicate, with independent replicates performed three times on different days.

### Measurement of metabolic activity of treated biofilms

To assess the viability of treated biofilms, a resazurin-conversion assay (Sigma-Aldrich, Ireland) was employed. A stock solution of resazurin (440 µM) was diluted to 88 µM in sterile water, and 100 µL of this solution was added to wells containing the biofilms. The plates were then incubated for 1 h at 37°C in the dark to allow for the conversion of resazurin by metabolically active cells. Subsequently, the fluorescent intensity of the wells was measured using a fluorimeter, Perkin Elmer 2030 Multilabel Reader Victor X3, with excitation at 544 nm and emission at 590 nm. The fluorescence intensity, indicative of biofilm metabolic activity and proportional to the number of living cells, was evaluated.

### Assessment of biofilm viable cells by colony count

After treatment, the biofilms established in the wells underwent two washes with 200 µL of PBS to remove any non-adherent cells. Subsequently, 100 µL of TrypLE solution (Gibco, Dublin, Ireland) was added to each well to detach the biofilm. Serial 10-fold dilutions were prepared in PBS from the detached biofilms, and 10 µL aliquots were plated in triplicate on MH agar using the drop method. The plates were then incubated at 37°C overnight and CFUs were enumerated, and the results were expressed as log_10_ CFU/mL.

### Biofilm formation using a biofilm flow device

For biofilm formation using a biofilm flow device, the colony count method was employed following the protocol established by Duckworth et al. (25). The Duckworth biofilm flow device was assembled, comprising twelve 10 mm agar disks (1.5%, wt/vol) prepared using a French press punch and placed into designated wells. Each agar disk was covered with a cellulose filter (13 mm, 0.22 µm, Merck Millipore, Ireland) and inoculated with 10 µL of bacterial suspension (10^8^ CFU/mL). The device was then incubated at 37°C with a continuous flow of SWF at a rate of 0.322 mL/min for either 24 h or 5 days, facilitated by a peristaltic pump (Fig. S1). Following incubation, a topical treatment with CHAPK-SH3blys was administered for an additional 6 h. The resulting biofilms on the cellulose filters were collected and suspended in 1 mL of PBS, and colony counting was performed.

Additionally, to facilitate confocal microscopy post-treatment, the Duckworth biofilm flow device was set up similarly to the colony count method with slight adjustments. Twelve agarose disks were prepared by dissolving 0.3 g of agarose in 20 mL of SWF supplemented with 3% fetal bovine serum (FBS) and 0.15% collagen (Merck, Ireland), mixed with bacterial cultures at 10^8^ CFU/mL, and cut into 10 mm disks. These agarose disks were then placed into designated wells in the Duckworth device and subjected to the same experimental conditions as described earlier. Post-treatment, thin slices of the agarose disks were cut and stained using the bacterial viability LIVE/DEAD BacLight kit (ThermoFisher Scientific, Ireland) according to the manufacturer’s instructions. The stained discs were imaged using a confocal laser scanning microscope (Cell Observer Z1, Zeiss, Oberkochen, Germany) equipped with a 63× objective. Image acquisition and processing were conducted using the Zeiss software package and ImageJ (ImageJ/Fiji 1.46, National Institutes of Health, USA), respectively, with three representative images obtained per sample group for each experiment.

### Investigation of the potential of agents to induce antimicrobial tolerance *in vitro*

Potential for the development of antimicrobial tolerance *in vitro* was investigated according to established protocols with certain modifications (26). In brief, the *in vitro* serial passage study involved exposing bacteria, diluted to a concentration of 10^4^ CFU/mL in cation-adjusted Mueller Hinton Broth (CAMHB), to CHAPK-SH3blys at half the previously determined bactericidal concentration. Serial passaging commenced by harvesting bacterial cells growing at sub-MIC concentration, inoculating them into fresh CAMHB with CHAPK-SH3blys, and re-incubating them for 18 h at 37°C. This process was repeated daily for a period of 14 days. The assay was performed in independent triplicates to ensure the reliability of the results.

### Effect of CHAPK-SH3blys on mammalian cell viability

The effect of CHAPK-SH3blys on mammalian cell viability was evaluated using three different cell lines: HaCaT cells (human keratinocyte cell line), THP-1 cells (human acute monocyte leukemic cell line), and HUVEC cells (human primary endothelial cells). HaCaT cells were cultured in Dulbecco’s modified Eagle’s medium (DMEM) supplemented with 10% fetal bovine serum (FBS), THP-1 cells were cultured in RPMI 1640 medium with 10% FBS, and HUVEC cells were cultured in EGM Bulletkit Endothelial cells medium supplemented with 10% FBS. Cells were seeded into the wells of a 96-well microtiter plate at a density of 10^5^ cells/mL. Controls were treated with Triton X-100 (1%, vol/vol) or medium alone. Subsequently, 500 µg/mL of 3-(4,5-dimethylthiazol-2-yl)−2,5-diphenyltetrazoliumbromide (MTT) in PBS was added to each well, followed by incubation for 4 h. After incubation, the wells were washed with PBS, and an MTT fixative solution (DMSO) was added. The plates were then fixed for 5 min with shaking, and the absorbance was measured at 595 nm using a PerkingElmer 2030 Multilabel Reader Victor X3. All incubations were carried out at 37°C in a 5% CO_2_ humidified incubator. Experiments were performed in triplicate, with independent replicates conducted three times on different days.

### Hemolysis assays

Potential hemolytic activity of CHAPK-SH3blys was assessed using fresh human erythrocytes obtained from healthy donors, following a method outlined by Cantisani et al. (27). Human blood samples were collected in ethylenediaminetetraacetid acid (EDTA) solution (1.6 mg/mL), and erythrocytes were isolated by centrifugation at 1,000 × *g* for 5 min at 18°C. After three washes with PBS, the erythrocytes were diluted twofold (vol/vol) in PBS and 100 µL aliquots were added to wells of 96 well plates. CHAPK-SH3blys was then added to the erythrocyte suspension at concentrations ranging from 3.125 to 200 µg/mL in a final volume of 200 µL. After incubation at 37°C for 24 h, solutions were centrifuged and the degree of hemolysis was determined by measuring the absorbance of the supernatant at 490 nm.

### Investigating the immune response to endolysin CHAPK-SH3blys

Whole blood obtained from healthy donors was incubated with either CHAPK-SH3blys or medium alone and one of the four bacterial strains tested for 4 h at 37°C in a 96-well microtiter plate in the presence of one of the tested bacterial strains (ThermoFisher, Ireland). Treated blood samples were collected into microfuge tubes and centrifuged at 150 × *g* for 10 min. Serum was decanted and stored at −20°C until further analysis. A comprehensive Cytokine Multiplex Panel 9-plex array (AssayGenie, Ireland) of proinflammatory and anti-inflammatory cytokines and chemokines (including interleukin-1 alpha and beta, IL-2, IL-4, IL-6, IL-8, IL-10, tumor necrosis factor-alpha [TNF-α], and interferon-gamma [IFN-γ]) was employed for cytokine profiling. The preparation of the assay plate adhered to the manufacturer’s instructions to ensure accuracy and consistency of the experimental procedure.

### Statistics

The data are presented as the mean ± SD. Statistical analyses were performed using GraphPad Prism version 9. Tests used for the *P*-value determination are specified in each figure legend.

## RESULTS

### CHAPK-SH3blys endolysin exhibits potent antimicrobial activity against *S. aureus*

**Fig 1:**
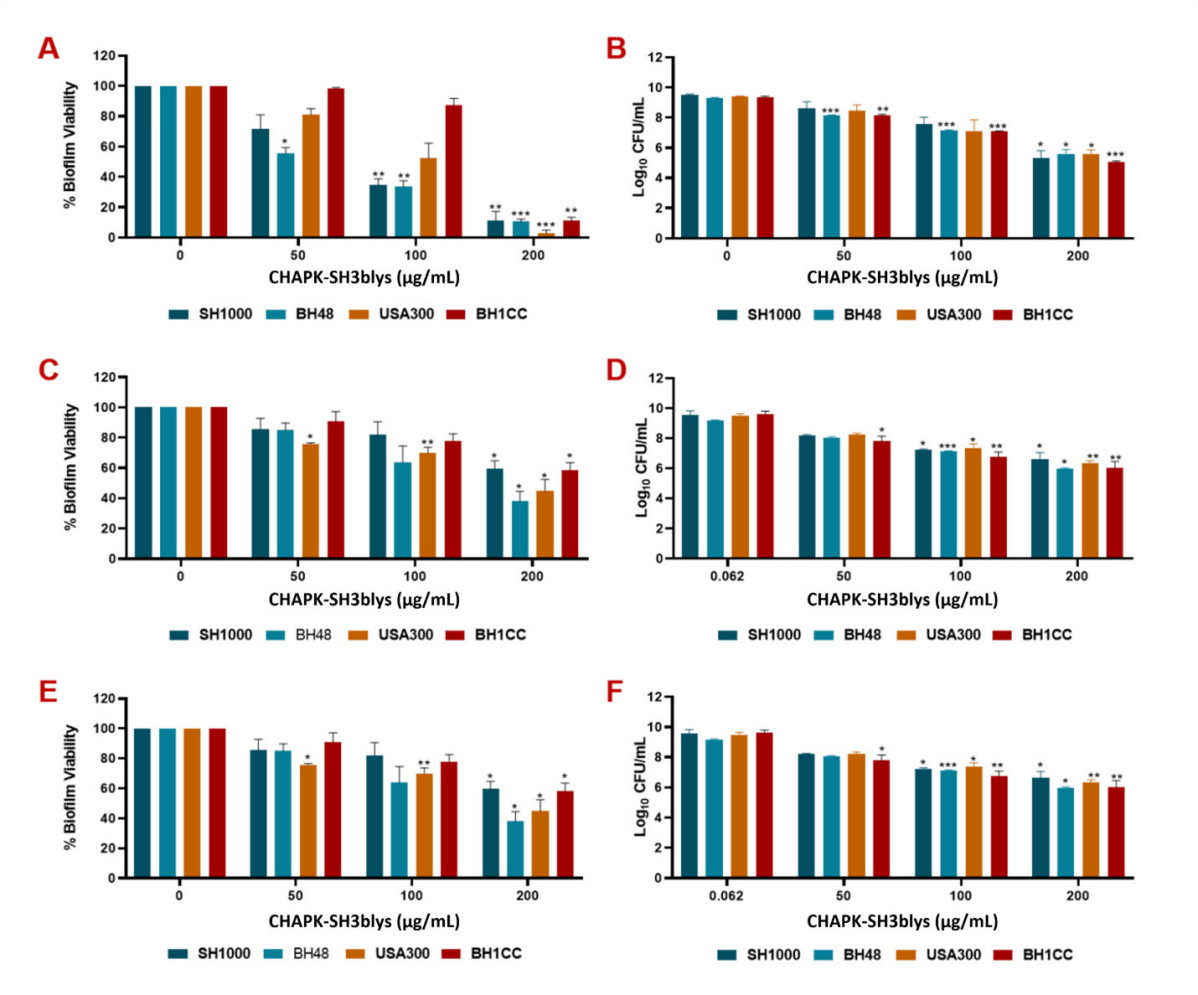
Efficacy of CHAPK-SH3blys free in solution against early and mature biofilms of *S. aureus*. Biofilms were formed for 1 day (**A, B**), 3 days (**C, D**), and 5 days (**E, F**) under*in vivo* like*in vitro* conditions before treating them with increasing concentrations of CHAPK-SH3blys for 1 h at 37°C. Biofilm viability was detected and presented as mean ± SD of three independent experiments. Data were analyzed using two-way ANOVA, and statistical differences were significant compared to the untreated control where *, **, and *** indicate a *P <* 0.05, 0.01, and 0.001, respectively*.*

In this work, CHAPK-SH3blys demonstrated potent antibacterial activity against clinical and reference strains of *S. aureus* with an MIC of 3.9 µg/mL (Table 1). Negative control experiments showed that the enzyme had no effect against PAO1. Conventional antibiotics tested including Fusidic acid, gentamicin, and mupirocin were shown to be effective against *S. aureus* strains while only gentamicin demonstrated activity against PAO1(Table 1). Given its low MICs against *S. aureus*, CHAPK-SH3blys antibiofilm activity was evaluated against preformed biofilms of the representative bacterial strains under static and dynamic conditions, as well as within an *in vivo*-like wound environment. Higher concentrations than the MIC were needed to disrupt biofilms, and a concentration range of 50–200 µg/mL, representing between 12 and 50 times the MIC, was used under static conditions. Treatment of 1, 3, and 5 day old static *S. aureus* biofilms for 2 h with CHAPK-SH3blys resulted in a concentration-dependent decrease in biofilm metabolic activity to approximately 80% (Fig. 1A), and up to a 4 log reduction in viable biofilm cell counts for 1 day biofilms at the highest concentration tested (Fig. 1B). Significant antibacterial activity was also evident against 3 day (Figure C, D) and 5 day (Figure E, F) biofilms albeit at a reduced level compared to 1 day biofilms.

**TABLE 1:** Minimum inhibitory concentrations (MIC) of the tested antimicrobial agents against reference and clinical strains of *S. aureus* and *P. aeruginosa*

	MIC µg/mL			
	CHAPK-SH3blys	Fusidic acid	Gentamicin	Mupirocin
MSSA SH1000	3.9	0.06	0.25	0.03
MSSA BH48	3.9	4	0.25	0.03
MRSA USA300	3.9	0.03	0.5	0.06
MRSA BH1CC	3.9	2	32	2
*P. aeruginosa* PAO1	>512	>512	1	>2,048

**Fig 2:**
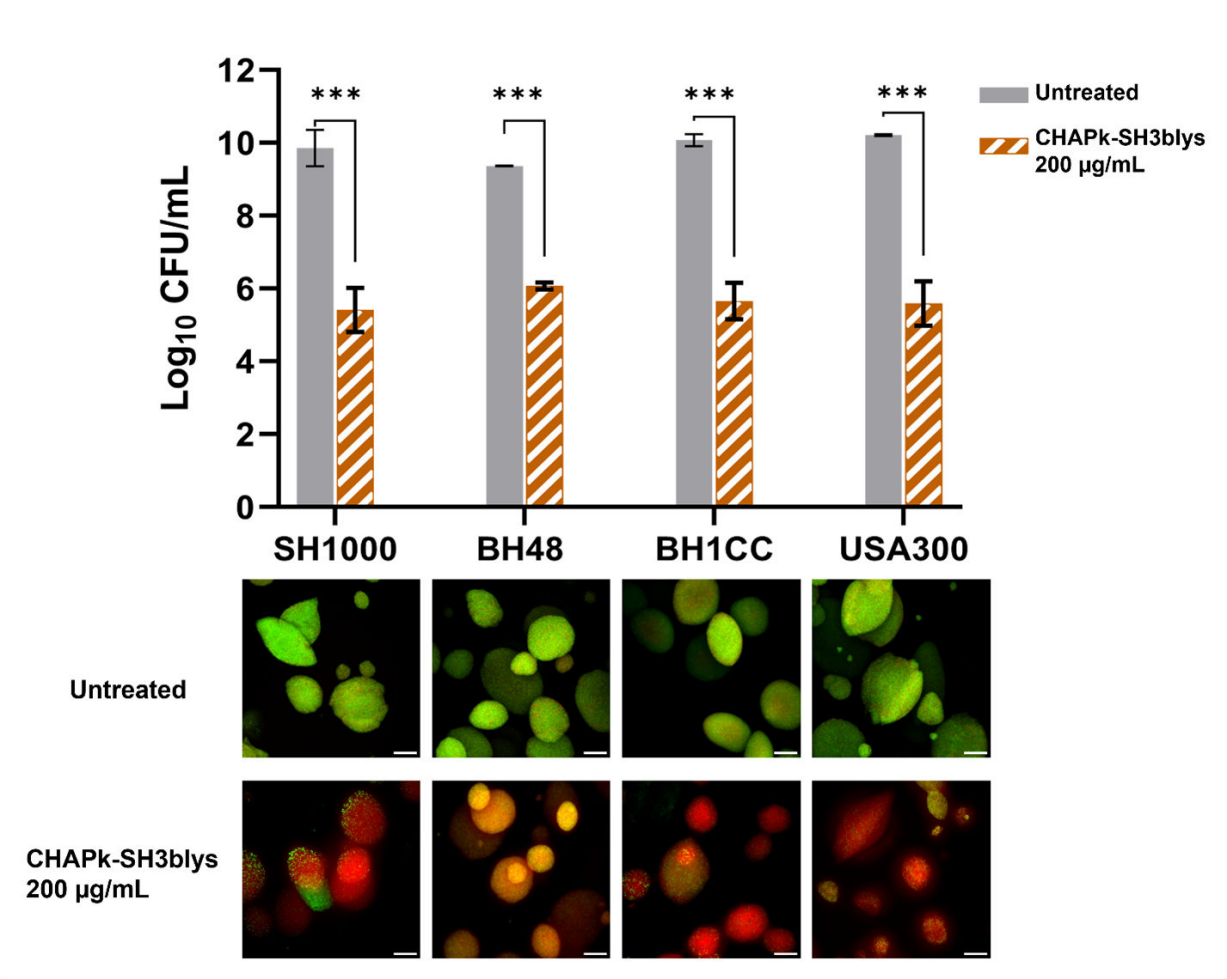
Antibiofilm activity of CHAPK-SH3blys against biofilms of *S. aureus*. Biofilms were allowed to grow for 5 days in Duckworth system before treating them with CHAPK-SH3blys. The graph shows a log10 CFU/mL ± SD in biofilms before and after treatment. Statistical tests of significance between treated and untreated biofilms were performed using Student’s *t*-test, where *** indicates *P* ≤ 0.001. Images represent confocal microscopy data of treated and untreated biofilms (Green-stained cells correspond to viable cells and red-stained cells correspond to dead bacterial cells). Observations were shown to be reproduced in three independent experiments. Scale bar corresponds to 100 µm

The Duckworth biofilm flow device was then utilized to further validate the efficacy of the endolysin under hydrodynamic shear conditions. The bacterial strains were cultured to form biofilms over 1 and 5 days within the Duckworth device, after which they were treated with 200 µg/mL of CHAPK-SH3blys for 2 h. Quantitative analysis of the outcomes revealed up to a 4-log reduction in CFU/mL compared to untreated controls (Fig. 2). Consistent with the quantitative data, biofilms examined using confocal microscopy (live/dead staining) revealed a marked increase in dead cells (red) relative to live cells (green) following endolysin treatment (Fig. 2). These data reveal the effectiveness of CHAPK-SH3blys in eradicating established *S. aureus* biofilms under static and shear flow conditions using wound infection models.

### CHAPK-SH3blys has low cytotoxic and hemolytic activity

**Fig 3:**
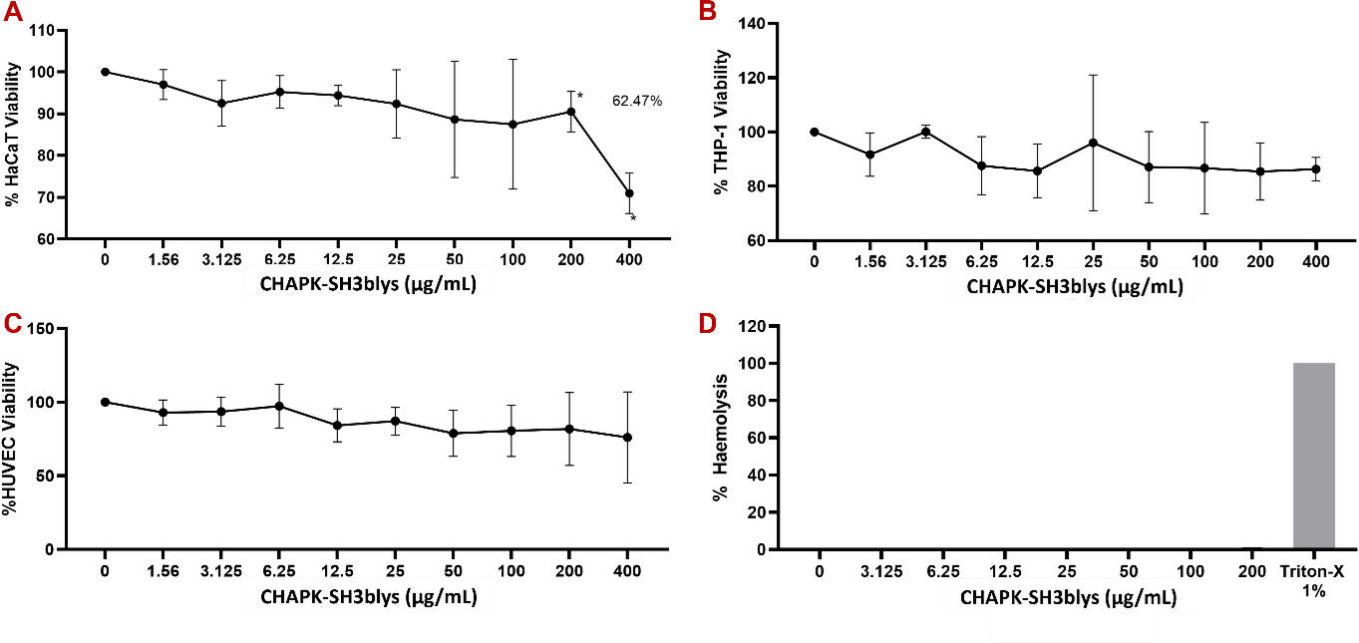
Effect of CHAPK-SH3blys on the cellular viability of human cell lines. Cells of (**A**) HaCaT, (**B**) THP-1, (**C**) HUVEC, and (**D**) Red blood cells were exposed to the endolysin for 24 h at 37°C, 5% CO_2_. Cell viability was assessed by MTT assay for **A, B**, and **C **while Absorbance was used to detect hemolysis activity in **D**. Data represent the average of three independent experiments (± SD). Where no value is present, a zero % effect is observed. Data were analyzed using one-way ANOVA test.

To evaluate the safety profile of CHAPK-SH3blys endolysin, investigations were conducted using human cell lines and human blood samples obtained from healthy donors. MTT assays was performed to determine *in vitro* cytotoxic effects of the endolysin on HaCaT (Fig. 3A), THP-1 (Fig. 3B), and HUVEC (Fig. 3C) cells in culture. Results revealed minimal cell cytotoxicity, with cell survival rates remaining comparable to untreated controls for THP-1 and HUVEC. At very high concentrations (200 and 400 µg/mL), CHAPK-SH3blys caused around 10% and 40% reduction in cell counts, respectively. Additionally, CHAPK-SH3blys had negligible hemolytic effects on human red blood cells from healthy donors (Fig. 3D). These data demonstrate the favorable safety profile of potential topical application of CHAPK-SH3blys.

### CHAPK-SH3blys endolysin does not induce an immune response or promote resistance development

**Fig 4:**
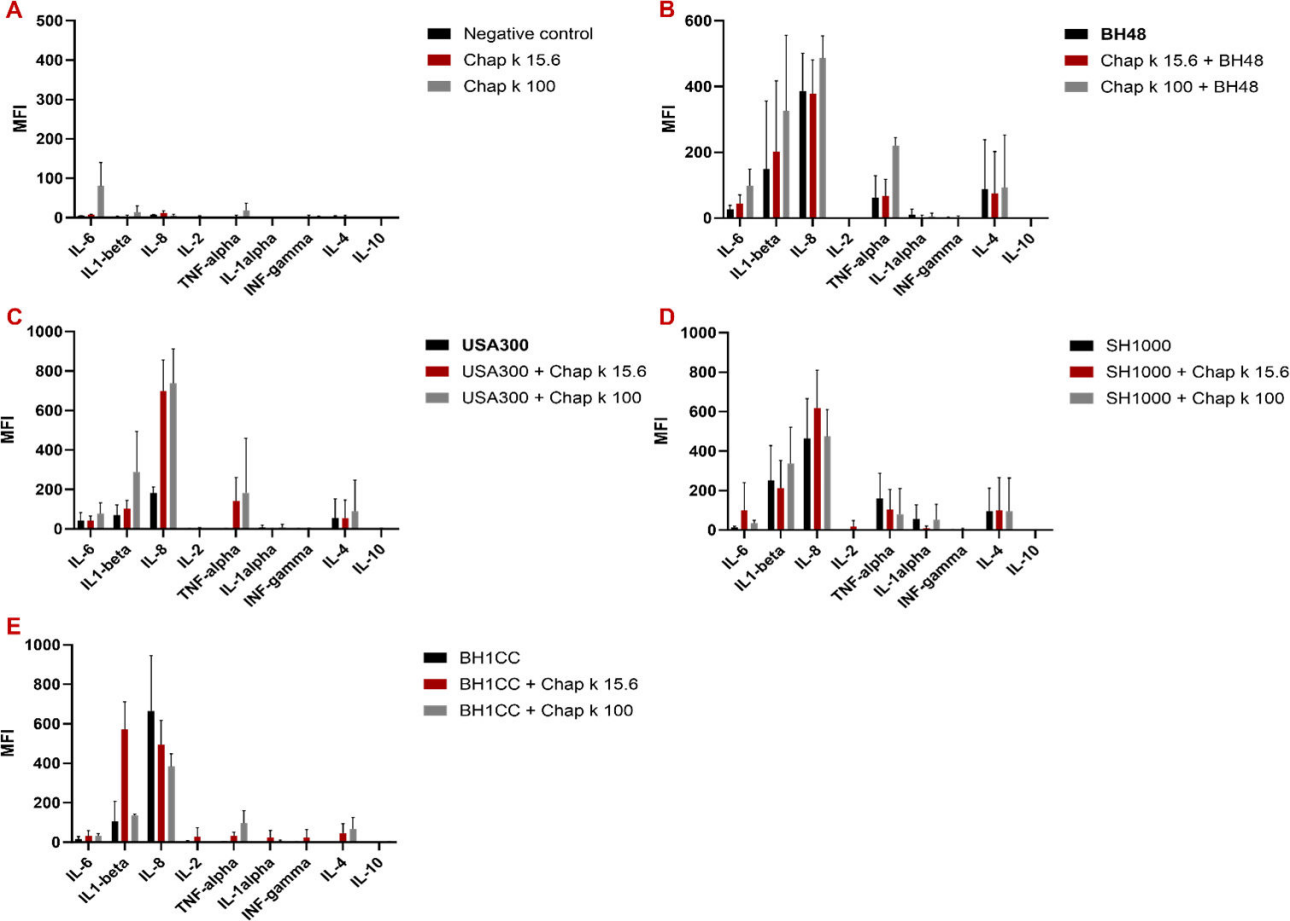
Effect of CHAPK-SH3blys on cytokines levels in human serum. Human blood was incubated with endolysin with or without the bacterial strains. (**A**) No bacteria, (**B**) BH48, (**C**) USA300, (**D**) SH1000, and (**E)** BH1CC for 4 h before centrifuging the cells and obtaining the serums. A multiplex assay was then used to detect the level of cytokines in each sample. The data represent the Mean fluorescence intensity (MFI) values (± SD) for each tested cytokine as detected by flow cytometry. The tests were performed three times for each sample.

**Fig 5:**
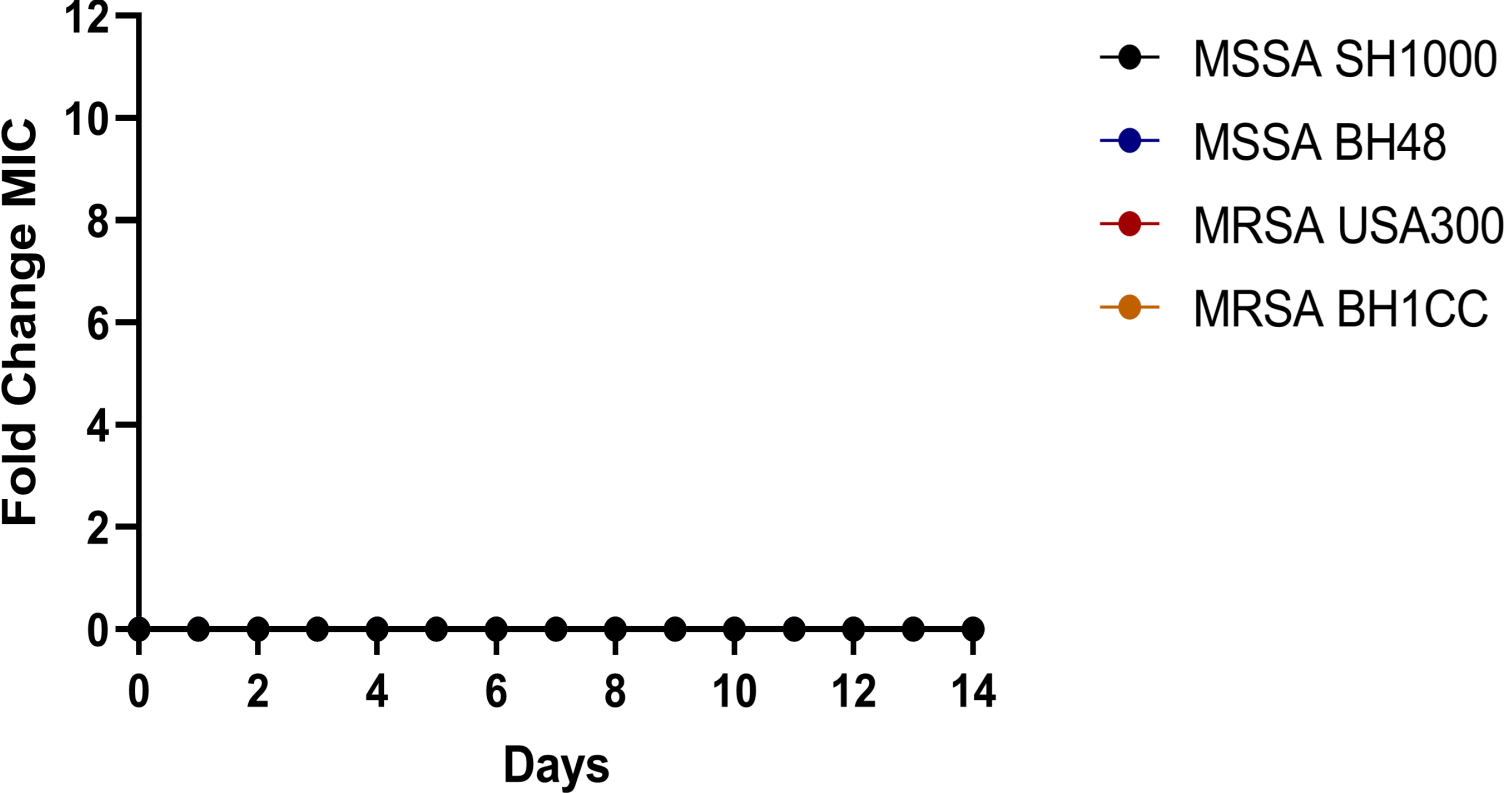
Assessment of the development of resistance to CHAPK-SH3blys over 14 sequential passages. The data represent the fold change in MIC of the endolysin after each passage over 14 days duration.

Cytokine induction assays were performed to assess the impact of endolysin on inflammatory markers. The selected cytokines have critical roles in mediating immune responses. The proinflammatory cytokines IL-1α, IL-1β, TNF-α, IL-6, and IL-8 were included to evaluate potential activation of the inflammatory pathways, which could indicate adverse immunostimulatory effects of CHAPK-SH3blys. Anti-inflammatory cytokines like IL-4 and IL-10 were measured to determine whether CHAPK-SH3blys might influence the regulatory components of the immune response. In these experiments, human blood was incubated with the endolysin alone (Fig. 4A) or with *S. aureus* strains BH48 (Fig. 4B), USA300 (Fig. 4C), SH1000 (Fig. 4D), and BH1CC (Fig. 4E). Overall CHAPK-SH3blys did not induce cytokine production, suggesting that it does not trigger an inflammatory effect and has no adverse impact on the tested cytokines. This further supports its suitability for use in human applications, as it is unlikely to provoke an immune response. Moreover, antimicrobial resistance testing demonstrated that continuous exposure of BH48, USA300, SH1000, and BH1CC to CHAPK-SH3blys at sub-MIC concentrations did not result in the emergence of resistance as the MIC remained unchanged after several passages (Fig. 5). This indicates that CHAPK-SH3blys maintains its effectiveness without encouraging resistance in target bacteria, underscoring its potential as a promising therapeutic agent.

## DISCUSSION

The treatment of wound infections has become increasingly challenging due to the increasing number of patients with chronic wounds involving bacterial biofilms within the wound bed and the rise of multidrug-resistant (MDR) bacteria (28). Traditional antibiotics are becoming less effective, prompting a search for alternative systemic and topical antimicrobial treatment options (29). One such treatment option is bacteriophages which are gaining attention due to their ability to target MDR pathogens, their stability in various environments, and their low side effects profile (30, 31). The enzymes produced by bacteriophages, namely endolysins, are able to degrade the bacterial cell wall, leading to cell lysis (32, 33). Their enzymatic nature allows them to act rapidly and effectively against both planktonic bacteria and those in biofilms (33) and are, therefore, being increasingly investigated as novel antimicrobial treatment options (34–36).

In a previous endolysin study by Horgan et al. (37), using the native staphylococcal endolysin LysK and various truncated derivatives, the deleted 165 amino acid derivative, subsequently designated CHAPk, was reported to have twofold higher activity against live cells than the native endolysin. Later, Arroyo-Moreno et al. (19), compared CHAPk for its biofilm disruption capacity with CHAPk-SH3blys. All concentrations of CHAPk-SH3blys produced a significantly higher biofilm disruption compared to the same concentrations of CHAPk when *in vitro* biofilms were induced from log-phase and 7-day-old cultures. Moreover, 7-day-old cultures produced stronger and more persistent biofilms, confirming findings that could already be seen in another study by Savijoki et al. (38). While both CHAPk and CHAPk-SH3blys were shown to prevent and disrupt biofilms formed from either log-phase cultures or 7-day-old cultures, CHAPk-SH3blys was more effective than CHAPk and, thus, chosen for this study.

The results presented in this study validate the potent and selective antimicrobial activity of CHAPK-SH3blys endolysin against *S. aureus*. The host range of CHAPk has been previously determined to be restricted to the *Staphylococcus* genus (37). It has demonstrated comparable efficacy against several species, including *S. aureus*, *S. caprae*, *S. haemolyticus*, *S. hyicus*, *S. hominis*, *S. chromogenes*, *S. saprophyticus*, *S. capitis*, and *S. epidermidis*, while exhibiting minimal activity against *Micrococcus luteus* and members of the *Streptococcus* genus. This highlights the treatment potential of CHAPk in confirmed staphylococcal wound infections, mainly in chronic wounds where biofilm formation is prevalent and *S. aureus* is the primary pathogen. In this study, the MIC of CHAPK-SH3blys endolysin was determined to be 3.9 µg/mL. This efficacy was observed across both clinical and reference strains, underscoring the broad applicability of CHAPK-SH3blys against this clinically significant pathogen. The specificity of CHAPK-SH3blys for Gram-positive bacteria, with no observed effect on *P. aeruginosa*, suggests a targeted mechanism of action, likely mediated by its affinity for peptidoglycan structures unique to Gram-positive cell walls, as shown in several studies on bacteriophages (39, 40). This specificity is a critical advantage, as it reduces the risk of off-target effects on commensal flora, which are often a concern with broad-spectrum antibiotics.

The antibiofilm activity of CHAPK-SH3blys was demonstrated under various conditions that mimic *in vivo* infection models, including static, dynamic, and *in vivo*-like wound environments containing blood. Biofilms represent a significant challenge in treating bacterial infections due to their resistance to conventional antibiotics and the protective matrix they produce (2), which can, however, be overcome and disrupted by endolysins (41, 42). Targeting biofilms typically necessitates significantly higher concentrations, often several-fold greater than the MIC. In this study, biofilms were grown over 1, 3, and 5 days to simulate early and mature biofilms, reflecting the clinical scenarios often encountered in patients with chronic wounds. The results demonstrated a concentration-dependent reduction in biofilm metabolic activity and viability, with up to a 4-log reduction in viable cell counts at the higher concentration tested which resembles 50 times the MIC. The Duckworth biofilm flow device was employed to further validate these findings under dynamic, wound-like conditions, where CHAPK-SH3blys showed a substantial decrease in biofilm viability. The importance of mimicking real wound conditions to improve clinical relevance has been well documented (43, 44). The substantial reduction in biofilm viability observed with CHAPK-SH3blys treatment highlights its potential as an effective agent against biofilm-associated infections.

The safety profile of CHAPK-SH3blys was thoroughly assessed, with no significant cytotoxicity observed in human cell lines, as determined by the MTT assay. Additionally, the absence of hemolytic activity in human red blood cells from healthy donors demonstrates that CHAPK-SH3blys does not disrupt cellular membranes or cause lysis of host cells, highlighting potential clinical applicability. Further supporting its safety for therapeutic use, CHAPK-SH3blys endolysin has shown to not induce a proinflammatory immune response, as demonstrated by the cytokine assay results. The importance of avoiding an immune response lies in reducing the risk of side effects that could arise from inflammation, such as tissue damage or systemic immune reactions, which are often associated with many antimicrobial treatments.

Moreover, we addressed the potential for antimicrobial resistance development during treatment. Continuous exposure to CHAPK-SH3blys at sub-MIC concentrations did not result in the development of bacterial resistance, suggesting that the endolysin retains its efficacy over time and may be less prone to resistance mechanisms that commonly affect traditional antibiotics. Unlike traditional antibiotics, endolysins have a different mode of action that targets essential components of the bacterial cell wall, making it difficult for bacteria to develop resistance. This resistance profile, combined with its potent antibiofilm activity and low toxicity, positions CHAPK-SH3blys as a promising therapeutic candidate for the treatment of *S. aureus* infections, particularly those complicated by biofilm formation.

In this study, we used revised models of infection that more closely mimic *in vivo* conditions, addressing limitations of earlier CHAPk studies. These models better reflect the clinical challenge posed by biofilms, which are highly resistant to antibiotics even at concentrations far exceeding the MIC (2, 45). Though the MIC value of the endolysin was higher than the tested antibiotics (Table 1), CHAPK-SH3blys offers distinct advantages. It effectively penetrates and disrupts biofilms, where conventional antibiotics often fail as shown in previous studies (45–47). Additionally, CHAPK-SH3blys offers a selective activity with reduced risk of resistance development. Despite higher concentrations, its antibiofilm activity makes it a promising alternative for treating biofilm-associated infections where antibiotics even at 50 times the MIC fail to be effective.

This study focused on *S. aureus* as the target organism, but the specificity of CHAPK-SH3blys limits its application to Gram-positive bacteria. While this specificity is advantageous for avoiding off-target effects, it underscores the need for combination therapies or additional agents to address polymicrobial infections involving Gram-negative bacteria. In summary, the findings from this study support the therapeutic potential of CHAPK-SH3blys endolysin as a targeted and effective treatment against *S. aureus*, including its biofilm-associated forms, with a favorable safety profile and minimal risk of resistance development. Further *in vivo* studies and clinical trials are warranted to confirm these results and to explore the full potential of CHAPK-SH3blys in treating *S. aureus* infections, particularly in complex cases involving biofilms.

## AFTER EPUB

*[This article was published on 5 August 2025 with an error in an author's current address. The current address was removed in the current version, posted on 13 August 2025.]*

## SUPPLEMENTAL MATERIAL

Fig. S1 spectrum.00716-25-s0001.docx Biofilm formation in duckworth device flow system.

ASM does not own the copyrights to Supplemental Material that may be linked to, or accessed through, an article. The authors have granted ASM a non-exclusive, world-wide license to publish the Supplemental Material files. Please contact the corresponding author directly for reuse.
