# Supplementary material for: Use of the bacteriophage-derived endolysin CHAPK-SH3blys as a potent novel treatment for biofilm-associated Staphylococcus aureus wound infections
Source: Microbiol Spectr. 2025 Aug 5;13(9):e00716-25. doi: 10.1128/spectrum.00716-25 (PMC12403842; doi:10.1128/spectrum.00716-25)
Supplement: Fig. S1 — Biofilm formation in duckworth device flow system. [file spectrum.00716-25-s0001.docx]

**Supplementary Material**


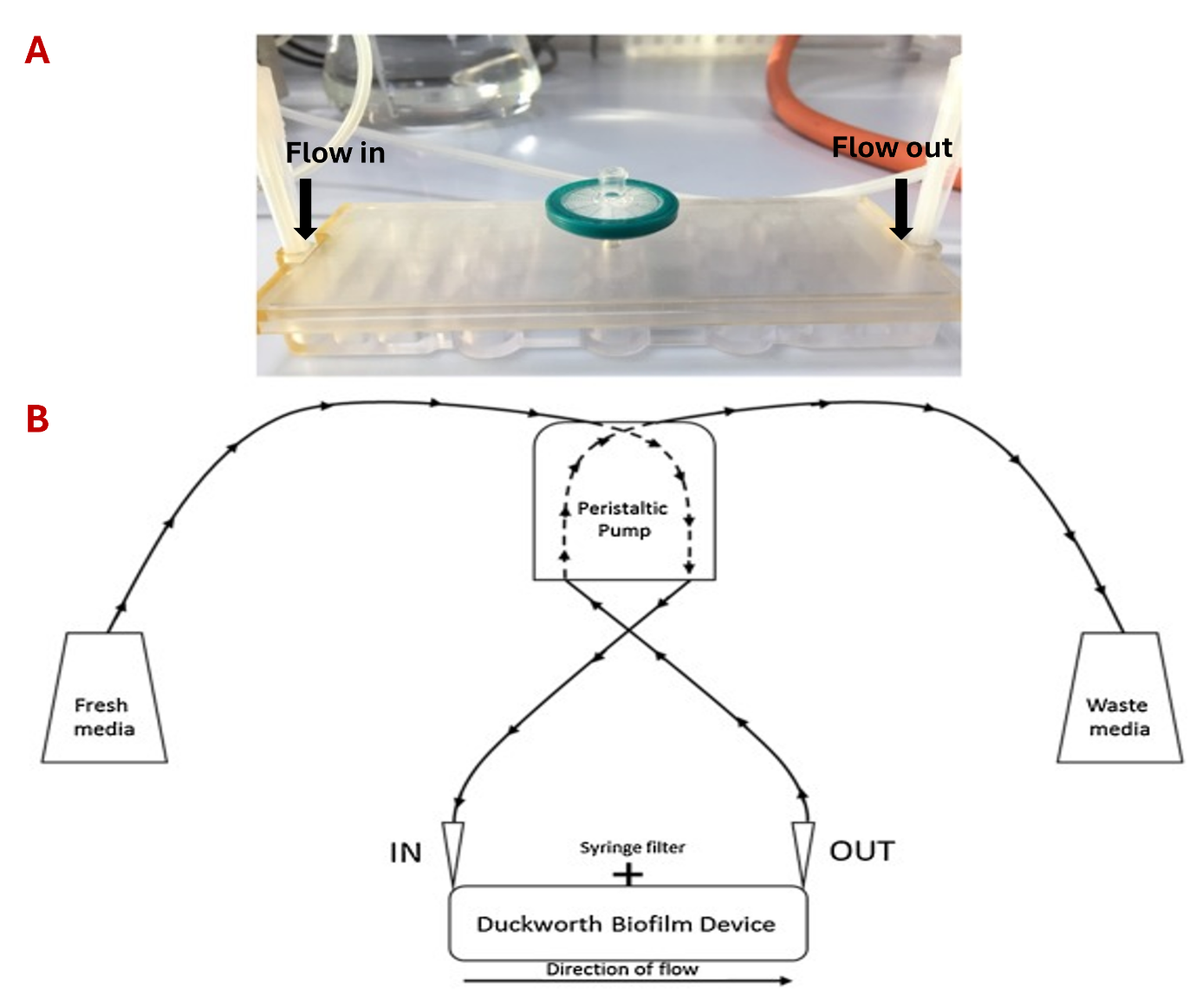


**Figure S1*.* Biofilm formation in duckworth device flow system.** A) Image showing the 3D printed Duckworth deivice and the connected system where the fresh media flow into the device and waste flows out. B)Ilustration of the duckworth system connections.
